# Targeting Human Glucocorticoid Receptors in Fear Learning: A Multiscale Integrated Approach to Study Functional Connectivity

**DOI:** 10.3390/ijms25020864

**Published:** 2024-01-10

**Authors:** Simone Battaglia, Chiara Di Fazio, Matteo Mazzà, Marco Tamietto, Alessio Avenanti

**Affiliations:** 1 Center for Studies and Research in Cognitive Neuroscience, Department of Psychology “Renzo Canestrari”, Cesena Campus, Alma Mater Studiorum Università di Bologna, 47521 Cesena, Italy; 2 Department of Psychology, University of Turin, 10124 Turin, Italy; 3 Neuropsicology and Cognitive Neuroscience Research Center (CINPSI Neurocog), Universidad Católica del Maule, Talca 3460000, Chile

**Keywords:** fear extinction, stress modulation, brain connectivity, glucocorticoid receptors, sex differences, anxiety disorders

## Abstract

Fear extinction is a phenomenon that involves a gradual reduction in conditioned fear responses through repeated exposure to fear-inducing cues. Functional brain connectivity assessments, such as functional magnetic resonance imaging (fMRI), provide valuable insights into how brain regions communicate during these processes. Stress, a ubiquitous aspect of life, influences fear learning and extinction by changing the activity of the amygdala, prefrontal cortex, and hippocampus, leading to enhanced fear responses and/or impaired extinction. Glucocorticoid receptors (GRs) are key to the stress response and show a dual function in fear regulation: while they enhance the consolidation of fear memories, they also facilitate extinction. Accordingly, GR dysregulation is associated with anxiety and mood disorders. Recent advancements in cognitive neuroscience underscore the need for a comprehensive understanding that integrates perspectives from the molecular, cellular, and systems levels. In particular, neuropharmacology provides valuable insights into neurotransmitter and receptor systems, aiding the investigation of mechanisms underlying fear regulation and potential therapeutic targets. A notable player in this context is cortisol, a key stress hormone, which significantly influences both fear memory reconsolidation and extinction processes. Gaining a thorough understanding of these intricate interactions has implications in terms of addressing psychiatric disorders related to stress. This review sheds light on the complex interactions between cognitive processes, emotions, and their neural bases. In this endeavor, our aim is to reshape the comprehension of fear, stress, and their implications for emotional well-being, ultimately aiding in the development of therapeutic interventions.

## 1. Introduction

The intricate interplay between cognitive and affective processes and the underlying neurobiological substrates has long attracted research in cognitive neuroscience [1]. Within the vast spectrum of human experience, fear emerges as a basic and evolutionarily conserved emotion, critical for survival [2,3]. Fear learning and fear extinction, essential for updating emotional associations and regulating behavior, have significant implications for the pathophysiology of anxiety disorders [4].

Understanding the neural substrates of these processes requires the exploration of functional brain connectivity, which involves the coordination and synchronization of neural activity among distinct brain regions [5]. The amygdala is a key subcortical structure playing a pivotal role in fear processing. Its connectivity with other brain regions, including the prefrontal cortex, is integral to regulating fear responses and contextualizing fear memories [6,7]. The prefrontal cortex exerts top-down control over emotional reactions and is crucial for cognitive processes involved in fear memory extinction [8,9,10]. The coordination between the amygdala and prefrontal cortex within functional brain networks is essential for adaptive fear regulation [4,11,12].

Fear learning is a fundamental learning process, deeply rooted in our evolutionary past, that helps us to predict potential threats [13,14,15]. It is a shared mechanism across species that allows individuals to rely on cues and environmental signals to anticipate danger and guide the selection of appropriate and secure responses tailored to the distinct characteristics of each species [14,16,17,18,19,20]. To investigate the neurobiological underpinnings of fear learning, researchers extensively examine humans and other species using Pavlovian classical conditioning paradigms [4,6,21,22]. These paradigms involve gradually associating a neutral stimulus (NS) with a threatening stimulus, commonly known as the unconditioned stimulus (US) [7,16]. By linking the NS with the US, the NS acquires then the ability to elicit a conditioned fear response, transforming it into a conditioned stimulus (CS+) [6,8,23]. 

These complex processes are controlled by a cortico-subcortical network involving the amygdala, which works together with prefrontal regions to form emotional memories [13]. The proper regulation of emotional responses to potential threats is pivotal in maintaining mental well-being, as disruptions in emotion regulation can contribute to trauma-related disorders, such as anxiety and mood disorders [7,18,24,25,26,27,28,29,30,31,32].

Fear extinction is an important counterbalance to enduring fear memory traces, relying on a dynamic interplay between the amygdala, prefrontal cortex, and hippocampus. Extinction involves reducing fear responses by exposing individuals to fear-inducing cues without consequences [14,26]. During fear extinction training, repeated presentations of the CS+ in the absence of the US lead to a reduction in the conditioned fear responses [33,34]. Extinction does not erase or unlearn memories; rather, it involves a new learning process where the cue is associated with the absence of the threatening event [35,36,37,38]. This new learning competes with the original CS–US association, influencing behavior during subsequent retention tests. Support for this interpretation comes from the observation of the spontaneous resurgence of extinguished fear memories [7,13,15,39,40,41,42,43]. Essentially, a new fear extinction memory restrains the CS–US association by updating the initial fear memory or suppressing the original memory trace, without erasing it during the extinction process (akin to relearning) [4,42,44,45,46]. These processes collectively contribute to the delicate balance between adaptive fear responses and maladaptive anxiety disorders [42,47].

It is important to consider that fear learning extends beyond memory consolidation, and it is inherently linked to the stress response. Stress is a multifaceted physiological and psychological response to perceived threats, challenges, or demands, associated with complex body changes, including an increased heart rate, elevated blood pressure, and heightened alertness [48,49,50,51,52,53]. These changes are designed to prepare the body to respond quickly to a potential threat and are mediated by the release of glucocorticoid hormones, such as cortisol. This hormonal release serves to prepare the organism to manage the source of stress by mobilizing energy, suppressing non-essential functions such as digestion or reproduction, and modulating the anti-inflammatory response [48,49,50,51,52,53].

Importantly, stress plays a role in memory formation [54,55,56] and can significantly impact fear learning and extinction [57]. Seminal studies have shown that stress influences the acquisition and retrieval of extinction in humans [58,59,60,61]. For instance, exposure to stress, such as through a cold pressor, can make it more difficult to extinguish a conditioned fear response, as seen in skin conductance responses (SCRs) [62]. Moreover, stress exposure before retrieval testing can impair the expression of extinction, resulting in the return of conditioned fear responses [61,63]. However, the influence of stress on learning is complex as moderate stress levels can potentially enhance neuroplasticity, whereas excessive stress could undermine it [64,65,66].

The hypothalamic–pituitary–adrenal (HPA) axis controls the body’s response to stress and triggers the release of glucocorticoids [48,49,50,51,52]. Central to the stress response are *glucocorticoid receptors* (*GRs*), which mediate the actions of glucocorticoids on target cells. These receptors are widely distributed across brain regions implicated in both fear learning and extinction [7,48,67]. Importantly, GRs play a bidirectional role in fear regulation: they enhance fear memory consolidation in the amygdala while facilitating extinction processes through their actions in the prefrontal cortex and hippocampus [53,68,69,70], in line with the notion that different levels of stress can have opposite influences on neuroplasticity [64,65,66]. Dysregulation of GR function has been implicated in the etiology of anxiety and mood disorders, underscoring the intricate relationship between stress, fear regulation, and emotional well-being [71,72,73]. The interplay between stress hormones, GRs, and fear regulation highlights the importance of maintaining a healthy balance in the stress response for optimal neuroplasticity and emotional well-being [64,65,66].

Experimental studies have employed synthetic glucocorticoids, such as *dexamethasone* (DEX), to regulate the HPA axis and reduce cortisol levels in the bloodstream; alternatively, other studies have incremented cortisol levels by directly administering 10–30 mg of cortisol, in order to elucidate the mechanisms through which cortisol exerts its effects [53,74,75,76]. These investigations contribute to understanding the molecular signaling pathways that play a role in how cortisol affects the consolidation, reconsolidation, and extinction of fear memories. Understanding these mechanisms is crucial not only in gaining insights into fear-related processes but also in developing potential therapeutic interventions in fear-related disorders [77,78,79,80,81,82,83].

Recent advancements in neuroscience have highlighted the need for a comprehensive understanding of fear learning and extinction, integrating molecular, cellular, and systems-level perspectives. Neuropharmacology, as a multiscale approach, offers a unique lens through which the intricate interplay of neurotransmitters, neuropeptides, and receptor systems can be examined [84,85]. This approach allows researchers to explore the effects of pharmacological manipulations on fear regulation, shedding light on the molecular underpinnings of fear-related disorders [83,86,87,88]. Moreover, neuropharmacological studies in neuroscience offer insights into potential therapeutic targets that could alleviate the debilitating effects of excessive fear. Post-traumatic stress disorder (PTSD) stands as an example of the profound ramifications of disrupted fear learning, extinction processes, and the stress response. In PTSD, there is a notable alteration in GR sensitivity, which contributes to an altered stress-related response and heightened inflammatory processes [71,89,90]. This dysregulation underscores the intricate link between stress, inflammation, and fear-related psychopathologies, highlighting the cascading effects of stress-related disruption on mental health [91,92,93,94,95,96,97].

The combination of cognitive neuroscience and neuropharmacology in the study of fear learning, fear extinction, and stress offers a unique exploration of human emotions. Hence, this review aims to explore the intricate pathways linking fear-related processes, brain connectivity, stress, GRs, and neuropharmacological investigations [84]. By embracing a multidimensional perspective, we aim to elucidate the interplay between stress and the formation of emotional memory, providing novel insights into fear-related disorders and potential therapeutic interventions.

## 2. Understanding the Effects of Glucocorticoids on Fear Learning

The effects of glucocorticoids, such as cortisol in humans, on brain regions involved in fear extinction have garnered significant attention due to their crucial role in shaping emotional learning and responses. Glucocorticoids, stress hormones primarily produced by the adrenal glands, have been found to modulate these fear extinction brain regions both directly and indirectly [48,98,99]. Directly, GRs are densely expressed in the amygdala, influencing its activity and the processing of fear-inducing stimuli [100,101,102]. Indirectly, the HPA axis, under glucocorticoid control, regulates fear extinction by affecting the prefrontal cortex and hippocampus [7,99]. Glucocorticoids impact this region by affecting its neuronal structure and functional connectivity; similarly, the hippocampus, facilitating context-dependent memory, is influenced by glucocorticoids, thus playing a pivotal role in fear extinction [103,104]. The effects of glucocorticoids on neuronal structures can be adaptive or maladaptive, depending on the context in which they are acting. For instance, glucocorticoids can modulate neurotransmission, synaptic plasticity, and the activation of various cell types in the nervous system, including neurons, glial cells, and microglia [105,106]: this modulation can contribute to learning, but it can also lead to maladaptive changes that contribute to neuropsychiatric disorders and neuropathic pain. Moreover, glucocorticoids can influence the expression of genes related to neuronal development and function, leading to changes in the maturation and connectivity of neurons [107]. This can have long-lasting effects on brain function and may increase the susceptibility to certain diseases, particularly when there is excessive glucocorticoid exposure during critical periods of brain development, affecting brain plasticity. Excessive glucocorticoid levels can also indirectly and negatively impact the amygdala, prefrontal cortex, and hippocampus, as shown in chronic stress [54,55,108,109]. In this section, we discuss studies examining the intricate relationship between glucocorticoids—primarily cortisol—fear extinction, and memory processes. To enhance the clarity and readability, we have categorized these studies based on the specific fear paradigms that they employ to explore the relevant research within their area of interest, whether it be fear extinction, fear renewal, and reinstatement paradigms or stimulus-based extinction generalization paradigms.

Different studies have investigated the complex relationship between glucocorticoids, including cortisol, and fear memory extinction. To evaluate the complex interplay between stress hormones and memory processes, Cornelisse et al. [110] explored the impact of cortisol on the consolidation of fear memories through a fear learning experiment that involved both delay and trace conditioning. In trace conditioning, the CS and the US are presented in sequence, but with a temporal gap or “trace” between them. This requires the subject to maintain a stable mental representation of the CS during the temporal gap in order to form the association with the US. Participants were divided into three groups (i.e., rapid cortisol administration, slow cortisol administration, and a placebo group), with the goal of manipulating cortisol levels to resemble those associated with acute stress. The participants’ physiological responses were measured using SCR and electromyography (EMG) of facial muscles. An electric shock was used as US, and participants’ expectations of shock intensities were continuously rated during different experimental phases. Neutral faces were presented as conditioned stimuli and the participants’ responses were measured during the acquisition, extinction, and reinstatement phases. The findings of the study revealed that both rapid and slow cortisol administration effectively increased cortisol levels during the designated phases, as cortisol intake before fear acquisition enhanced trace fear conditioning but did not affect the delay fear paradigm. The analysis of the delay and trace conditioning paradigms indicated that the conditioning phases were successful in eliciting physiological responses associated with fear. Additionally, when investigating the effects of rapid and slow cortisol administration, the study found that neither approach significantly impacted baseline SCR responses or responses during CS presentation in the acquisition phase. The analysis of the extinction phase revealed that the slow cortisol group showed more differentiation of the trace stimulus compared to the placebo group, indicating the enhanced fear memory of the trace stimulus 24 h later, as evidenced by the enhanced startle responses during early extinction. However, the effects on SCR and expectancy ratings were not significant. These suggest that slow (gene-mediated) corticosteroid effects during fear acquisition can strengthen subsequent fear memory in humans, particularly in the case of trace conditioning. Moreover, the effect on trace conditioning is likely mediated by genomic effects during fear conditioning and early consolidation, which were not present in the rapid cortisol group. In conclusion, this study showed how cortisol, administered shortly before fear acquisition, can influence the consolidation of fear memories. Specifically, it appears to enhance the strength of fear memories in the context of trace conditioning.

In a similar study, Merz et al. [111] sought to investigate the influence of cortisol on fear learning and the subsequent extinction process, focusing on both physiological and neural responses. Participants underwent the fear learning procedure, which involved pairing a CS+ (i.e., grayscale images of a rhombus and a square) with a US (i.e., electric stimulation), or the presentation of the CS alone (CS−). The study also assessed participants’ cortisol levels: some participants were administered a hydrocortisone dose, while others received a placebo. The cortisol concentrations were analyzed, and participants were asked to guess whether they had received cortisol or a placebo. SCRs were recorded as indicators of physiological arousal in response to the CS+ and CS− stimuli during both the acquisition and extinction phases. Functional magnetic resonance imaging (fMRI) was used to examine neural responses and brain regions of interest (ROIs), including the amygdala, anterior cingulate gyrus, nucleus accumbens, hippocampus, and medial frontal and orbitofrontal cortex. The results revealed differential physiological and neural responses during fear acquisition and extinction: participants exhibited higher SCRs to the CS+ compared to the CS−, and this differentiation decreased over time during both acquisition and extinction. Importantly, cortisol administration appeared to influence the extinction phase: cortisol reduced conditioned SCRs, indicating a decrease in physiological fear responses. Additionally, cortisol time-dependently diminished conditioned responding, as evidenced by less differential SCRs in the early blocks of extinction. Furthermore, cortisol altered the time course of extinction learning in the amygdala, anterior parahippocampal gyrus, hippocampus, and insula. It also influenced the functional connectivity of the anterior parahippocampal gyrus with the ventromedial prefrontal cortex (vmPFC). Overall, cortisol administration resulted in a neural activation pattern likely reflecting stronger inhibitory processes, without generalizing to the behavioral level. The authors showed that cortisol administration impacts both physiological and neural responses, particularly during the later stages of extinction.

In a subsequent study of the same research group, Merz et al. [64] examined the effects of cortisol administration before extinction training on fear extinction. The study involved three days of experiments. Participants underwent acquisition training in context ‘A’ on the first day, extinction training in context ‘B’ on the second day, and recall in context ‘B’ and a new context ‘C’ one week later. They were conditioned to images representing different rooms: lamps of different colors were used as CSs, while the US was an electrical stimulation. Cortisol was administered 50 min prior to the start of the extinction training phase on the second day, to assess how it might influence fear extinction processes and memory recall. The research methodology involved analyses of SCRs and fMRI data, displaying neural activation and functional connectivity during the extinction training. Notably, the findings showed that cortisol administration led to a time-dependent reduction in conditioned responding during extinction training, and this was evidenced by less SCRs in the first and second blocks of extinction training in the cortisol group compared with the placebo group. Cortisol also significantly altered the time course of extinction learning in the bilateral amygdala, the right anterior parahippocampal gyrus, and the right hippocampus, and demonstrated an influence on the functional connectivity between specific brain regions associated with the process of fear extinction. Finally, the results showed that cortisol had a context-dependent effect on extinction memory, which aligned with the observed reduction in SCRs and diminished amygdala–hippocampal complex activation during extinction learning when cortisol was administered. This suggests that cortisol may facilitate the consolidation of extinction memory within the context in which it is learned, rather than generalizing to a new context. The results also showed that the cortisol group had reduced SCRs compared to the placebo group during the recall phase, which supports the idea that cortisol can have a lasting impact on memory consolidation. Moreover, the findings that cortisol diminished SCRs and activation of the amygdala–hippocampal complex during extinction learning indicate that cortisol may change the recall of extinction memory by altering its consolidation. However, these effects were restricted to the extinction context, as cortisol did not modulate conditioned responding in a new context. This is consistent with the observation that cortisol-treated participants exhibited higher SCRs toward the CS + E (extinguished stimulus) compared with CS− during recall in context B, suggesting that cortisol’s effects on extinction memory did not generalize to new contexts.

Likewise, the work of Kinner et al. [112] aimed to study the mechanisms underpinning the return of fear (ROF) and the effects of cortisol administration on fear reinstatement. Their study focused on the role of cortisol in the retrieval of fear memories, investigating how cortisol administration impacts fear responses during distinct phases of the fear learning process, specifically extinction, renewal, and reinstatement. The fear learning paradigm, which took place on two consecutive days, involved the use of photos of two different rooms as contexts, with different colored desk lamps serving as CSs. The US was an aversive electrical stimulation delivered during fear acquisition. The participants underwent fear acquisition and extinction on the first day and renewal and reinstatement tests on the second day. The renewal test involved presenting the CSs in both contexts without any electrical stimulation, while the reinstatement test included the unsignaled delivery of the US. Neurophysiological methods, including SCRs to measure conditioned fear responses, were employed to assess participants’ fear responses to different stimuli. Additionally, cortisol administration and fMRI were used to investigate fear responses and neural activity during fear learning and extinction, as in the previously discussed studies. Results showed that during the renewal test, stronger differential activation of the left orbitofrontal cortex was found in the acquisition context ‘A’ compared to the extinction context ‘B’, potentially representing the neural response of fear renewal. This was observed without modulations by treatment or sex. In contrast, during the context-dependent reinstatement test, the authors found that cortisol enhanced fear responding, particularly in the originally safe extinction context ‘B’, indicating an inability to use contextual information to express extinction memories. Furthermore, cortisol-treated men exhibited higher differential SCRs in context ‘B’ compared to men who received a placebo. However, this increase was not observed in women. Cortisol also impaired the contextualization of fear memories, resulting in fear generalization to other CSs and contexts. Interestingly, the fMRI analysis revealed differential neural activations during renewal and reinstatement tests. These activations were influenced by cortisol administration and sex, with different patterns of activation observed in men and women: in men, cortisol treatment led to increased activation of the amygdala during the reinstatement of fear, suggesting greater fear responses [113,114]. This pattern suggested reduced amygdala activation in women under cortisol administration, potentially indicating a blunted fear response compared to men [111,115,116]. Cortisol specifically amplified the ROF after re-exposure to unsignaled US (reinstatement) in men, which was characterized by an enhanced differential amygdala response in context ‘B’ compared to context ‘A’, showing that glucocorticoids negatively impact extinction memory, ultimately leading to a stronger return of fear in men.

Shifting the focus to stimulus-based extinction generalization, Hagedorn et al. [117] investigated the neural underpinnings of stimulus-based extinction generalization, a strategy with potential implications in terms of enhancing exposure therapy. The experimental design spanned three days, encompassing phases of fear learning, extinction training, and recall. The central focus was investigating the impact of various CSs, as well as cortisol administration, on fear-related processes. The fear learning phase featured a paradigm with three distinct geometric shapes functioning as CS, and the US was an electrical stimulation. The successful fear acquisition was indicated by SCRs as well as activation observed in regions of the brain associated with fear, such as the amygdala, insula, and dorsal anterior cingulate cortex (dACC). During the subsequent extinction training phase, participants were exposed to the CS + G (generalized) and CS + N (non-generalized) stimuli: while the CS + N were presented in their original size, the CS + G were presented in three smaller sizes (75%, 50%, and 25% of the original size) in addition to the original size, to test for generalization effects. Interestingly, as the training progressed, there was a decrease in activation within the fear network, encompassing the amygdala, insula, and dACC. This reduction in activation was more pronounced during the second half of extinction training. However, no significant differences emerged between the responses to the CS + G and CS + N stimuli, suggesting comparable extinction learning for both types of conditioned stimuli. Moreover, in the recall phase, participants’ SCR and neural activation patterns showed no differences between the CS + G and CS + N stimuli. This finding underscored the success of the stimulus-based extinction generalization approach, implying that the learning from the extinction training effectively generalized to different stimuli. To investigate the potential role of stress in this context, the researchers administered hydrocortisone tablets to half of the participants—with the aim of mimicking stress effects—while the other half received placebos, in a randomized double-blind manner. The cortisol administration was successful, as evidenced by higher cortisol levels in the treatment group. The most notable observation in the cortisol manipulation phase was the interaction between the factor of cortisol administration and fear learning phases. In the extinction learning phase, cortisol seemed to affect neural responses to the CS + G. This was evident as the amygdala and insula showed increased activation, while the vmPFC showed decreased activation, compared to classical extinction learning, suggesting that extinction generalization could heighten arousal and enhance novelty or salience during the learning process. On the other hand, during the recall phase, there was deactivation in the amygdala and parahippocampal gyrus activation, along with increased functional connectivity to the hippocampus. This could indicate a reduction in fear expression or less emotional memory recall in response to the CS + G compared to the non-generalized stimuli (CS + N). This neural pattern was blocked by cortisol administration; in the placebo group, there was a decrease in amygdala and insula activation and reduced functional connectivity with the vmPFC for the CS + G compared to the CS + N. In contrast, no difference was observed between the CS + G and CS + N in the cortisol group. Finally, while cortisol did not significantly affect the overall pattern of extinction generalization, it did interact with neural responses, potentially influencing the differentiation between CS + G and CS + N.

In a follow-up study [114], the same research group further explored the impact of cortisol administration on fear extinction. The study involved fear learning procedures using geometric shapes as CSs paired with electrical stimulation used as US to elicit fear responses. Additionally, fMRI was employed to elucidate the neural mechanisms underlying these processes. Cortisol administration took place on the second day of the study, and salivary cortisol levels were measured as a physiological marker of stress. SCRs were used as physiological indicators of fear responses during fear learning and extinction training. Cortisol administration successfully elevated cortisol levels. Indeed, in the second half of extinction training, CS + G induced increased activation in the bilateral insula and dACC compared to both CS− and CS + N. SCR revealed enhanced responses to both CS+ and CS− during the initial half of extinction training. The retrieval phase showed reduced activation in the left anterior hippocampus for CS + G (generalized extinction) vs. the CS + N (standard extinction), with cortisol modulating this effect. SCR indicated elevated responses to both CS+ and CS− during retrieval. The reinstatement test displayed decreased activation in the left amygdala and dACC for CS + G relative to CS + N, influenced by cortisol. However, SCRs did not exhibit significant differences between CS+ and CS−. Moreover, cortisol did not significantly affect extinction training but appeared to influence neural responses during retrieval and reinstatement, potentially reducing fear-related processing for the CS + N. Overall, the study highlights the complex neural mechanisms involved in extinction generalization and suggests that cortisol may play a role in modulating these processes, particularly during memory retrieval and reinstatement.

Finally, Brueckner et al. [118] explored the effects of cortisol on fear learning, extinction, and the subsequent ROF among a group of healthy, non-smoking students. Participants were assigned randomly to either the cortisol or placebo group. The fear learning procedure unfolded over a three-day timeline, comprising the acquisition training, extinction training, and the ROF test. At each phase, participants were exposed to distinct neutral facial images portraying both male and female individuals, namely CS+ and CS−. These were paired with either distressing (US) or neutral film clips to elicit specific fear responses. Meanwhile, physiological measures, like SCR and fear potentiated startle (FPS), were recorded, and the participants’ reactions underwent a comprehensive assessment. FPS was evaluated by measuring the electromyographic response of the orbicularis oculi muscle to loud sounds, eliciting the eye-blink reflex. As part of the experimental design, participants in the cortisol group were administered cortisol pills immediately after the extinction training. In contrast, those in the placebo group received placebo pills. Those in the cortisol group exhibited a distinctive pattern of CS+ expectancy ratings before the acquisition, which subsequently diminished after the acquisition phase. This pattern differed from the placebo group. Both groups displayed a reduction in fear responses during the extinction phase, indicative of some level of extinction learning. Interestingly, cortisol influenced both US expectancy, an explicit measure of the appraisal of the likelihood of a subsequent threat, and FPS, an implicit measure of threat expectancy linked to the amygdala’s influence on startle circuits. However, the cortisol group showcased enhanced extinction learning, as evidenced by their more robust SCR and FPS responses during this phase. In the ROF test, participants who received cortisol exhibited distinctive responses compared to those in the placebo group. Specifically, the cortisol group displayed reduced fear responses to the CS+ relative to the placebo group. This discrepancy suggests that cortisol administration could mitigate the resurgence of fear in response to the CS+.

**Figure 1:**
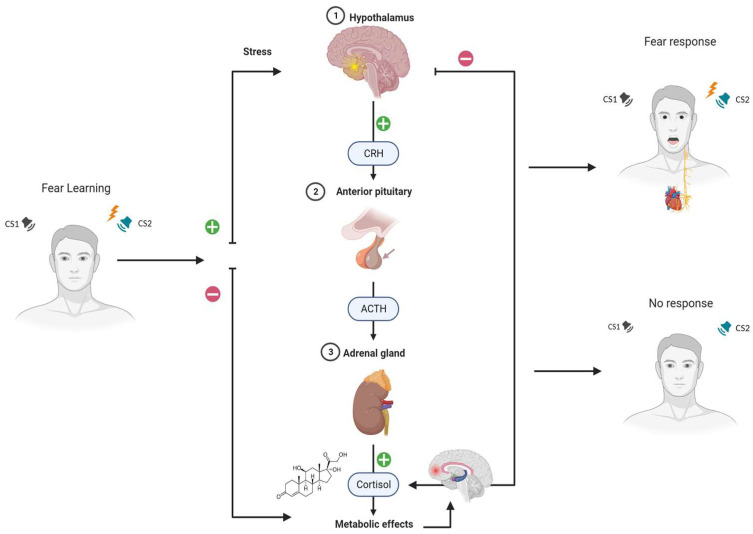
**Modulation of glucocorticoids on the HPA axis and fear learning**. Fear learning involves the process by which a neutral stimulus becomes threatening when associated with an aversive one, leading to conditioned fear responses. This process is regulated by a specific neural network, primarily involving the amygdala, prefrontal cortex, and hippocampus. In the context of emotional learning and fear responses, glucocorticoids, including cortisol, exert direct and indirect effects on brain regions involved in emotional learning and fear responses. Specifically, cortisol modulation can enhance fear memory consolidation, influence neural responses during extinction and reinstatement phases, and reduce fear responses to conditioned stimuli. In particular, during fear learning, the HPA axis becomes activated. The HPA axis is responsible for regulating the body’s response to stress and is involved in the release of cortisol, a stress hormone produced by the adrenal glands. Notes. CS1 = Conditioned Stimulus 1; CS2 = Conditioned Stimulus 2; CRH = corticotropin-releasing hormone; ACTH = adrenocorticotropic hormone.

In conclusion, these studies provide valuable insights into the impact of cortisol on fear-related physiological and brain responses and suggest that cortisol administration can enhance fear memory consolidation and extinction (see Figure 1). Elevated glucocorticoid levels have been associated with amygdala hyperactivity, impaired prefrontal cortex function, and hippocampal alterations, all of which hinder fear extinction. While these studies enhance our understanding of cortisol’s impact on fear-related processes, the intricate mechanisms of glucocorticoids in influencing brain regions during fear extinction remain complex and warrant further exploration (see Table 1).

## 3. Glucocorticoids and Gender Influence on Fear Extinction

As previously mentioned, glucocorticoids exert a profound impact on fear extinction, a process that is influenced by stress responses. The interplay of these hormones, integral to the HPA axis, manifests in circadian rhythms and stress-induced bursts [120,121]. The impact of glucocorticoids on memory processes is time-dependent: their secretion pre- or immediately post-learning enhances consolidation, while post-learning secretion hampers retrieval [122,123]. Particularly relevant to emotional memory, glucocorticoid activation augments consolidation; despite the typical attenuation of emotional memories over time, disorders like PTSD can perpetuate their intensity [98]. Sex hormones and gender distinctions exert significant influences on fear extinction, with women showing greater susceptibility to anxiety disorders and PTSD and gender disparities evident in emotional learning [124,125,126]. Sex hormones, influenced by factors such as menstrual cycle shifts and hormonal contraceptives, further complicate this picture [127,128]. Nonetheless, research often prioritizes male participants, emphasizing the need to elucidate the role of sex hormones in fear extinction [124,129,130]. The HPA, which is responsible for stress-induced activation and the release of glucocorticoids, plays a significant role in influencing cognitive processes, including the effects of stress and cortisol on memory, especially for emotional material, as consistently demonstrated in research [98,131]. A thorough understanding of cortisol, sex hormones, and emotional memory’s interplay is pivotal, holding promise in enhancing our comprehension of stress-related disorders and refining therapeutic strategies. Researchers have long been intrigued by ways to help individuals to overcome debilitating fears. One avenue of exploration is the hormone cortisol, which plays a pivotal role in the body’s stress response. Additionally, studies have shown that certain brain receptors, particularly NMDA receptors, are key players in forming and erasing fear memories [132,133].

In a pivotal study, Stark et al. [66] explored how cortisol might impact fear learning and extinction and how it might interact with gender differences. Participants were divided into four distinct groups based on gender and treatment: female placebo, female cortisol, male placebo, and male cortisol. The study design employed a double-blind, placebo-controlled approach. Some participants received 30 mg of cortisol orally, while others received visually identical placebos. The fear learning task utilized simple geometric figures, serving as CS+ and CS−, and electrical stimulations as US. SCRs were monitored simultaneously with fMRI scans. These responses were analyzed to understand fear reactions and potential differences between the groups. Interestingly, when looking at fear responses, distinct patterns emerged between males and females. Cortisol seemed to impact fear responses differently based on gender. When participants were exposed to conditioned stimuli, fMRI scans revealed stronger activations in brain regions associated with fear processing. This suggests that cortisol has differential effects on fear conditioning: specifically, the study observed that, in men, stress exposure facilitated fear conditioning, while, in women, stress appeared to inhibit fear conditioning. Additionally, these findings highlighted the distinct impact on neural responses in males and females, particularly in prefrontal regions, including the anterior cingulate, lateral orbitofrontal cortex, and medial prefrontal cortex.

To further investigate the interplay between hormonal factors and fear learning mechanisms, Merz et al. [134] decided to conduct a study on how cortisol affects neural activity and SCRs during fear conditioning when participants are not aware of the contingencies between CS and US. Participants were divided into four groups: cortisol women, placebo women, cortisol men, and placebo men. The experiment consisted of an acquisition phase, an extinction phase, and a two-back task. The participants were conditioned using visual stimuli (CS+ and CS−) paired with electrical stimulation (US). The experiment aimed to assess fear acquisition, extinction, and contingency awareness: contingency awareness was evaluated using a recognition questionnaire, and only participants who were unaware of the CS–US relationship were included in further analysis. Cortisol levels were manipulated through the administration of cortisol tablets or placebos. SCRs were measured to assess fear responses. The researchers successfully manipulated cortisol levels, inducing elevated cortisol concentrations in the cortisol-administered group. However, these levels did not significantly impact the participants’ performance in the two-back task. SCR results revealed a sex-dependent response to cortisol, with women exhibiting reduced SCR responses and men showing slightly heightened SCRs after cortisol intake. Neuroimaging data demonstrated that, irrespective of the treatment and phases, both CS+ and CS− evoked differential brain responses. Men showed CS+ vs. CS− differentiation in the right frontal cortex, insula, and thalamus, while women exhibited differentiation in the left frontal cortex, right insula, and right thalamus. Interestingly, cortisol influenced this pattern differently based on sex, as women showed the enhanced differentiation of CS+ and CS− activation within these brain structures after cortisol administration, while men showed reduced differentiation. Cortisol’s influence on fear learning proves to be sex-dependent, enhancing the differentiation of brain activation patterns in women and diminishing such patterns in men.

In line with these findings, in a subsequent study [135], the same research group administered either cortisol or a placebo before participating in a fear learning protocol. The participants were divided into three sex hormone status groups: free-cycling women, women taking oral contraceptives (OC), and men. Free-cycling women were invited during the luteal phase of their menstrual cycle, while women taking monophasic oral contraceptives were included if they had been using the contraceptive for at least three months. The study encompassed a multifaceted approach, including hormone analyses, SCRs, and neuroimaging assessments. Cortisol levels were measured at specific time points, coinciding with placebo intake, immediately before and after the fMRI session. Saliva samples were collected and assayed for cortisol, estradiol, progesterone, and testosterone concentrations. Moreover, the study employed SCRs to gauge fear responses. In this fear learning experiment, CS+ were represented by three gray-colored geometric figures: a rhomb, a square, and a triangle. Among them, the triangle served as the non-CS or distractor stimulus. The US was a transcutaneous electrical stimulation delivered through electrodes attached to the participants’ left shin. The findings of this study highlighted intricate relationships between cortisol, sex hormones, and fear learning. Notably, cortisol levels showed variations over time, with significant differences among the sex hormone status groups. All groups exhibited a decline in cortisol concentrations from the first to the second sample: men had higher cortisol levels after the fear learning procedure compared to before, while women’s levels did not differ significantly. Additionally, estradiol, progesterone, and testosterone levels differed significantly among the sex hormone status groups. Free-cycling women had higher estradiol and progesterone levels compared to women taking oral contraceptives and men. Men had higher testosterone concentrations than women in both groups. Finally, a brain imaging analysis revealed significant activation responses during fear learning. The differences between CS+ vs. CS− and US vs. non-US showed substantial activations in brain regions associated with fear learning, such as the amygdala and insula. Moreover, distinct group differences emerged in the CS+ versus CS− contrast, specifically in the right amygdala. The findings contribute to our knowledge of how hormonal and stress-related factors influence emotional learning and fear processing: cortisol levels were positively associated with amygdala activation during fear conditioning, with differences observed between men and women. Men exhibited left amygdala activation, while women taking oral contraceptives showed right amygdala and right anterior parahippocampal gyrus activation. Moreover, elevated cortisol levels were associated with the enhanced acquisition and consolidation of fearful memories, but the specific effects varied depending on sex and hormonal status (as men and women on oral contraceptives seemed to be more affected by cortisol in this context).

Meir Drexler et al. [136] explored the relationship between cortisol and fear memory reconsolidation by investigating a group of male participants. The rationale for this gender-specific approach stemmed from the need to isolate cortisol’s effects from potential confounding factors such as sex hormones. Participants were divided into three groups: reactivation + cortisol (RE + CORT), reactivation + placebo (RE), and no reactivation + cortisol (CORT). The study design spanned three consecutive days, each with distinct phases. Day 1 marked the fear acquisition phase, during which participants encountered CS paired with mild electric shocks, establishing fear associations. Day 2 introduced the experimental manipulation involving cortisol administration and the reactivation of specific stimuli. On this day, participants in the reactivation groups were subjected to re-exposure to one of the previously reinforced stimuli (CS1+), either with cortisol or placebo administration, while the no-reactivation group experienced only pill intake. Day 3 witnessed extinction learning, followed by reinstatement and a final reinstatement test. The reinstatement test session lasted for 30 min, and 24-h breaks were used to allow memory consolidation after the learning phase. Participants’ physiological responses were monitored through SCRs during the acquisition, extinction, and reinstatement phases. Cortisol concentrations were tracked via saliva sampling to gauge the success of the pharmacological intervention. The visual cues employed CS+ consisting of three distinct geometric shapes, while, to elicit conditioned responses, mild electric shocks (US) were associated with CS presentations, creating a fear association. Cortisol concentrations experienced a significant surge in both the RE + CORT and CORT groups post-administration, underlining the hormone’s influence on the fear memory reconsolidation process. During fear acquisition, participants showcased heightened SCRs to CS+ in contrast to CS−, reinforcing successful fear association establishment. The extinction phase witnessed a decline in SCRs across all groups, indicating effective memory extinction. However, a notable difference emerged during the reinstatement test. In the RE + CORT group, the reactivated stimulus (CS1+) exhibited significantly higher fear reinstatement compared to the non-reactivated stimulus (CS2+). This effect was not observed in the other groups, indicating that the administration of cortisol during reactivation was linked to a heightened reinstatement response for the reactivated stimulus, offering new insights into the intricate workings of fear memory and the potential influence of stress hormones.

Based on these findings, the same research group [137] investigated the effects of cortisol administration on fear responses and ROF in women using the same methodology. The study included female participants who were randomly assigned to three distinct experimental groups: reactivation + cortisol (RE + CORT), reactivation + placebo (RE), and no reactivation + cortisol (CORT). During the acquisition phase, participants underwent a fear learning paradigm. They were presented with two geometrical figures that acted as conditioned stimuli (CS1+ and CS2+), which were reinforced with an electric shock (US) in approximately 70% of presentations. Another figure (CS−) was never reinforced. On day 2, participants in the RE + CORT and RE groups received either cortisol or a placebo. The reactivation groups (RE + CORT and RE) experienced memory reactivation by being attached to SCR electrodes and shock electrodes. The no-reactivation group (CORT) received no additional intervention. This phase aimed to induce a prediction error for reconsolidation processes. Finally, on day 3, extinction trials involved the presentation of all stimuli (CS1+, CS2+, CS−) without reinforcement. Reinstatement, intended to reactivate fear memories, included unsignaled US presentations. The subsequent reinstatement test evaluated the ROF responses for each stimulus. The results demonstrated that participants exhibited higher SCR responses to the reinforced CS+ compared to the unreinforced CS−. This indicated that the fear learning process was successful across all groups. Extinction blocks showed higher SCR responses to previously reinforced stimuli, which decreased as the extinction trials progressed. This suggested successful extinction learning across all groups. Finally, the reinstatement test revealed no group differences in the reinstatement of the three stimuli. Moreover, there were no significant differences in the strength of reactivated fear memory between the groups. The cortisol analyses confirmed that cortisol concentrations were significantly higher 30 and 45 min after treatment in the cortisol groups (RE + CORT and CORT) compared to the placebo group (RE). This indicated a successful pharmacological treatment. In conclusion, the study demonstrates that while cortisol administration influences fear learning and extinction processes, it does not significantly affect the strength of reactivated fear memories during the reinstatement phase.

Building upon these findings, Meir Drexler et al. [138] continued investigating the impact of cortisol and US reactivation on fear memory reconsolidation in male participants. The previous findings indicated that differences in cortisol effects on fear memory reconsolidation might exist between men and women. Hormonal influences, like those related to the menstrual cycle or hormonal contraceptives, were believed to contribute to these differences. To isolate these effects, the study focused solely on male participants. Seventy-five men participated in the study. They were divided into three groups: one group underwent US reactivation along with cortisol administration (RE + CORT), another underwent US reactivation with a placebo (RE), and the third group received cortisol without reactivation (CORT). The study used two geometrical shapes (square and rhombus) as CS+, while the US was an electric shock administered to the participants’ left shin. The experiment spanned three days. On day 1, participants underwent fear acquisition training, where CS+ was paired with the US, and CS− was not. Day 2 involved pharmacological treatment (cortisol or placebo) and UC reactivation. Day 3 included extinction training and a subsequent reinstatement test. Skin conductance responses (SCR) were used as indicators of conditioned fear. Saliva samples were collected to measure cortisol levels. Cortisol concentrations were significantly higher in the RE + CORT and CORT groups after the administration of cortisol, compared to the RE group, which received a placebo. Successful fear acquisition was evident as participants showed higher SCRs to the CS+ than the CS−. Fear extinction was successful, with reduced SCRs to CS+ in the late phase of extinction compared to the early phase. In the CORT group, a reinstatement effect was observed in the response to the original CS+. However, no such effect was seen in the RE or RE + CORT group. There was a general increase in SCRs in response to modified stimuli (CS+/M and CS−/M) after the reinstatement shocks, regardless of the group. The study revealed that cortisol administration and UC reactivation had a differential impact on fear memory reconsolidation. Cortisol enhanced fear memory reinstatement when the original stimuli were used, but this effect was not observed when modified stimuli were presented. 

**Figure 2:**
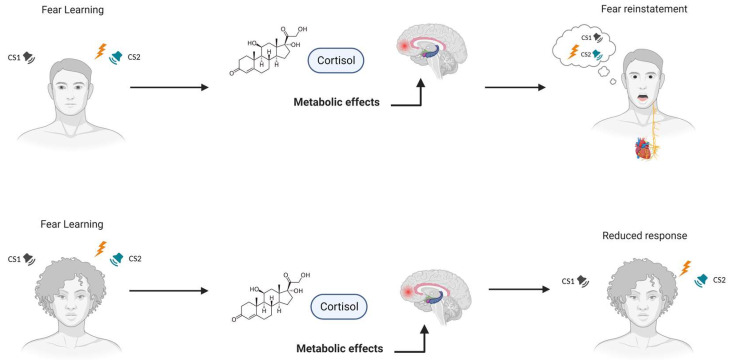
**Cortisol’s time-dependent impact on fear memory processes and gender-specific effects.** Glucocorticoids have a significant impact on fear extinction, which is influenced by stress responses and memory modulation. Cortisol exerts different effects on fear learning, also based on gender, particularly in prefrontal brain regions, as it influences prefrontal brain activation during the acquisition of fear learning in men. Moreover, cortisol has been shown to strengthen amygdala signaling in men, potentially increasing the return of fear and influencing fear-related brain regions. Furthermore, women exhibit greater susceptibility to anxiety disorders and PTSD, with sex differences observed in emotional learning and fear extinction responses. Notes. CS1 = Conditioned Stimulus 1; CS2 = Conditioned Stimulus 2; PTSD = post-traumatic stress disorder.

In summary, glucocorticoids exert time-dependent effects on fear learning, with pre- or immediately post-acquisition administration enhancing fear memory consolidation (see Table 2). These studies reveal distinct patterns of brain activation in response to cortisol administration, particularly within prefrontal regions associated with fear, thus highlighting that cortisol’s impact on fear conditioning extends beyond a one-size-fits-all paradigm and is influenced by gender. Furthermore, cortisol intake reduced the SCRs in women, indicative of diminished fear responses, while it slightly heightened the SCRs in men. These results were paralleled by differential neural activation patterns, further underscoring the gender-specific effects of cortisol on emotional memory processes. Cortisol emerges as a key player in enhancing amygdala activation during fear conditioning, showcasing pronounced gender-specific effects. Notably, its administration in males enhances fear memory reinstatement, particularly when employing the original stimuli. All in all, these studies collectively emphasize how the cortisol hormone can differentially influence fear responses in male and female individuals (see Figure 2).

## 4. Discussion 

Fear learning and fear extinction are complex psychological phenomena involving a gradual increase and then reduction in specific fear responses through repeated exposure [4,8,16]. Fear learning refers to a process that involves the brain’s ability to associate a neutral stimulus with a threatening or aversive event [16]. During fear learning, an individual undergoes repeated exposure to a CS, which is typically neutral, in conjunction with the US, which elicits a fear response [23]. Fear extinction is the subsequent phase, which involves reducing or inhibiting the conditioned fear response that was previously acquired during fear learning. It occurs through repeated exposure to the CS without the accompanying aversive event. During this phase, the individual learns that the CS no longer predicts the US [25,139]. This learning is often associated with a process of inhibitory learning, where the brain actively suppresses the fear response while not erasing the original fear memory [7,140]. Fear learning and fear extinction are intricate processes influenced by numerous factors, including genetics, individual differences, and the context in which the learning occurs [118,141,142,143,144,145]. The intensity of the fear-inducing stimuli, the emotional state of the individual, and even the level of stress and glucocorticoid hormones all play vital roles in determining the effectiveness of fear extinction [118,146].

Research has demonstrated that fear learning and extinction paradigms can be effectively used in animals and human adults to shed light on the complex interactions between cognitive processes, emotions, and their neural bases [147,148,149]. In animal studies, memory reconsolidation and extinction processes exhibit distinct signatures. Memory reconsolidation allows fear memories to be updated to a less aversive level through the incorporation of appetitive information [150]. This process involves the reactivation of consolidated memories, returning them to a protein-synthesis-dependent state: the temporal dynamics of memory reconsolidation are dependent on the strength and age of the memory, with younger and weaker memories being more easily reconsolidated than older and stronger ones [151]. On the other hand, extinction does not erase classically conditioned fear memories but can reduce fear in such a way that it does not return, consistent with a brain-wide modification of the original fear memory [152]. However, research has also shown that fear extinction is not always permanent, and fear can return under certain conditions, a phenomenon known as ROF [153]. This concept is supported by the understanding that extinction is a new learning process, and fear reduction results from the inhibition rather than the erasure of the original fear memory [154]. Therefore, gaining insights into the mechanisms and factors contributing to fear relapse is crucial in developing more effective treatments for anxiety disorders.

Stress, a complex response to perceived threats or challenges, can significantly impact fear learning and extinction [57]. When an individual is exposed to stress, the body releases glucocorticoid hormones, such as cortisol, as part of the stress response [53]. These hormones can modulate the consolidation and recall of fear memories, making it both more challenging to extinguish conditioned fear responses and more prone to fear relapse. In this regard, transitioning from clinical to animal studies in the study of stress and glucocorticoid modulation in fear learning provides valuable insights into the neurobiological mechanisms underlying fear regulation. Animal studies have been instrumental in highlighting the impact of stress and glucocorticoids on fear and extinction learning, anxiety, and trauma- and stressor-related disorders. Notably, using fear learning paradigms in mice, studies have demonstrated the stress-induced enhancement of fear learning, providing an animal model for PTSD [155]. Furthermore, animal evidence has shown that the modulation of glucocorticoids impacts fear extinction consolidation, highlighting the intricate interplay between stress hormones and fear regulation in animal models [156]. Animal studies have contributed to our understanding of the role of glucocorticoids in modulating the strength of memory for contextual learned fear [157], and these findings underscore the translational relevance of animal models in elucidating the molecular and cellular mechanisms underlying fear memory modulation by glucocorticoids. Furthermore, animal studies have revealed the regulatory role of glucocorticoids and norepinephrine in the formation of fearful memories in rodents and humans, emphasizing the importance of these stress-related mediators in memory processes [158].

In humans, recent studies have increasingly harnessed the relevance of brain plasticity and connectivity to dissect the dynamics of these neural networks during fear regulation [24,29,159,160,161,162,163,164], and novel non-invasive methods to strengthen neural pathways via plasticity induction appear particularly promising [165,166,167,168]. It is evident that stress significantly alters the connectivity patterns within these networks. Stress-induced changes in the activity of the amygdala, prefrontal cortex, and hippocampus can lead to heightened fear responses and impaired extinction, both of which are hallmarks of anxiety and mood disorders [169,170,171]. Thus, stress plays a pivotal role in influencing fear learning and extinction processes by altering the activity of key brain regions, including the amygdala, prefrontal cortex, and hippocampus. Dysregulation of these processes has been linked to enhanced fear responses and impaired extinction, contributing to anxiety and mood disorders [4,18,19,24,25,35,38,172,173]. GRs play a pivotal role in the stress response [174] and serve a dual role in fear regulation: they enhance the consolidation of fear memories while also facilitating extinction processes [175]. Dysregulation of GRs is associated with anxiety and mood disorders, underscoring their importance in emotional well-being [176,177].

In light of recent advancements in neuroscience, there is a growing emphasis on the importance of a comprehensive understanding that integrates perspectives from the molecular, cellular, and systems levels [178]. Neuropharmacology, in particular, provides valuable insights into the functioning of neurotransmitter and receptor systems, aiding in the investigation of mechanisms related to fear regulation and potential therapeutic targets [179,180]. Among these, cortisol, a key stress hormone, significantly influences both fear memory reconsolidation and extinction processes. A comprehensive understanding of these intricate interactions holds great promise in addressing psychiatric disorders related to stress [181]. The studies reviewed here provide valuable insights into the impact of cortisol on fear-related brain regions and responses, suggesting that cortisol administration can enhance fear memory consolidation and extinction, particularly in the context of trace learning [15,182], in line with the potential role of cortisol in neuroplasticity [64,65,66]. Despite these benefits, it is important to note that elevated glucocorticoid levels are associated with amygdala hyperactivity, impaired prefrontal cortex function, and hippocampal alterations, all of which can impair fear extinction processes [183,184]. The modulatory role of cortisol can be better understood by examining its effects on neural responses during the extinction, renewal, and reinstatement phases [178,180]. However, the intricacies of glucocorticoids’ mechanisms in influencing these neural responses and their effects on fear extinction are complex and require further exploration. Current evidence indicates that cortisol exerts time-dependent effects on memory processes, with pre- or immediately post-learning secretion enhancing consolidation and post-learning secretion hindering retrieval. Furthermore, the studies reviewed in this work reveal the gender-specific effects of cortisol on fear processing, highlighting distinct patterns of brain activation and emotional learning in men and women. These gender-specific effects underscore the nuanced interplay between hormonal factors and emotional memory processes.

It is crucial also to recognize that the exploration of the underlying mechanisms of PTSD points towards the intricate interplay between dysregulated fear responses and cortisol dysregulation [182,185,186]. Understanding these dynamics opens the door to potential clinical applications. The dysregulation of the HPA axis, cortisol dysregulation, pronounced hyperarousal responses, and impaired fear extinction mechanisms are recognized as pivotal factors in shaping the clinical features of PTSD. The enduring nature of this disorder and the limitations of established treatment modalities, like cognitive-behavioral therapy and pharmacotherapy [187,188], pose significant challenges, sparking interest in the development of innovative interventions.

A promising area of research focuses on the modulation of cortisol. The ability to decrease cortisol levels is of significant interest, especially in relation to enhancing fear extinction. This research should be embedded within a larger context that takes into account the intricate interactions between fear dysregulation, the HPA axis, cortisol modulation, and GR sensitivity. Investigating these interactions can deepen our understanding of susceptibility and resilience in traumatic experiences and could offer valuable insights into the potentials effects of synthetic glucocorticoids like DEX and other compounds on fear acquisition and extinction [189,190]. Moreover, recent advancements in neuroimaging have shed light on the significance of altered corticocortical connectivity patterns in conditions such as depression, anxiety disorders, and PTSD [191,192]. Functional connectivity changes have been linked to specific symptoms and even recovery during treatment in clinical populations. The complex and multifactorial nature of these mechanisms involves dynamic changes in glutamatergic signaling, synaptic strength, neurotrophins, cell adhesion molecules, and interactions with various neuromodulators [193,194]. Moreover, stress-related alterations in functional connectivity emerge from intricate interactions with genetic and neurodevelopmental factors that influence an individual’s susceptibility and resilience [191,195]. 

One significant avenue of investigation involves the impact of glucocorticoid stress hormones on dendritic remodeling and postsynaptic dendritic spine plasticity within vulnerable brain regions, including the hippocampus, prefrontal cortex, and amygdala [191,193]. Glucocorticoids, indeed, play a vital role in maintaining homeostasis. Recent research suggests that chronic stress disrupts glucocorticoid oscillations, which are crucial for synaptic remodeling, learning, and development [196]. Additionally, disruptions in structural and functional connectivity across distributed neural networks have been identified as common features of stress-related neuropsychiatric conditions [196,197]. These findings offer insights into the mechanisms of resilience and vulnerability, highlighting the remarkable neuroplasticity of brain networks and their capacity for recovery following stress exposure [198,199]. Nevertheless, individuals with neuropsychiatric disorders often exhibit persistent connectivity deficits even after stress cessation or treatment.

In conclusion, the comprehensive exploration of fear extinction, cortisol’s role in fear regulation, and the impact on mental health disorders such as PTSD offers a multidimensional perspective on emotional well-being. Collectively, the studies discussed in this review underscore the intricate relationship between cortisol, gender, and fear memory processes. This body of research enhances our understanding of how hormonal factors influence fear responses differently in males and females [114,135,200]. Moreover, the studies reviewed here contribute to our understanding of cortisol’s influence on fear memory consolidation and extinction, shedding light on its potential as a therapeutic target [187,188]. As the field of cognitive neuroscience continues to advance, the complex interplay between molecular, cellular, and systems-level processes in fear regulation becomes increasingly clear. This knowledge holds promise for the development of innovative interventions to address anxiety and mood disorders, ultimately improving the emotional well-being of individuals affected by these conditions.

Future research should continue to investigate the precise mechanisms through which cortisol modulates fear extinction and memory processes. Additionally, exploring the gender-specific effects of cortisol and other hormonal factors in greater detail can provide deeper insights into emotional learning and its implications for mental health. Innovative therapeutic approaches targeting the HPA axis, GRs, and cortisol regulation offer exciting avenues to improve the treatment of anxiety and mood disorders, including PTSD.

However, it is important to acknowledge and address the limitations inherent in these studies. The complexity of neurobiological interactions demands cautious interpretation, considering variables such as individual differences, diverse trauma experiences, and hormonal fluctuations, all of which introduce confounding factors [77,201,202]. The full extent of cortisol’s impact on human health remains not yet fully understood, underscoring the need for neuroimaging and molecular investigations to unveil its modulation mechanisms. Additionally, it is crucial to take into account the context that shapes our responses to fear and traumatic experiences. Looking ahead, the path that emerges from these studies presents new avenues of research, as unraveling the gender-specific dimensions of fear processing and cortisol modulation holds the promise of tailored interventions. Integrating advanced neuroimaging technologies and neurochemical assays may unveil the intricacies of cortisol’s interactions with specific neural networks. Furthermore, investigations into novel therapeutic approaches, potentially leveraging precision medicine and neuromodulation techniques, could significantly transform the landscape of fear-related disorders. These advancements signal a potential paradigm shift, emphasizing personalized treatments and the integration of cutting-edge technologies to enhance clinical outcomes and foster overall health and well-being.

## Figures and Tables

**Table 1:** Summary of findings in studies that explore the effects of cortisol on fear extinction and memory processes.

Study	Group (N)	Pharmacological Treatment	Mechanism of Action	Phase of Fear Learning	CSs and US	Psychophysiological Measure	Main Findings
Cornelisse et al. [110]	‘Slow cort’ (21) ‘Rapid cort’ (21) Placebo (21)	10 mg of cortisol Placebo	Cortisol is an agonist of glucocorticoid receptor and Annexin A1	Acquisition Extinction Reinstatement	CS: Male neutral faces US: Auditory tone	SCR-EMG	No effect of cortisol on SCRs ‘Slow cort’ group showed more differentiation of CS’s trace, compared to placebo group and ‘Rapid cort’ group
Merz et al. [111]	Cortisol (16) Placebo (16)	30 mg of cortisol Placebo	Cortisol is an agonist of glucocorticoid receptor and Annexin A1	Acquisition Extinction	CS: Geometric figures US: Electric shock	SCR-fMRI	Placebo group showed an enhanced reduction in SCRs from early to late extinction to the CS+, compared to the cortisol group Cortisol group showed diminished activation of the amygdala, MFC, and NAcc during late extinction
Merz et al. [64]	Cortisol group (20) Placebo group (20)	30 mg of cortisol Placebo	Cortisol is an agonist of glucocorticoid receptor and Annexin A1	Acquisition Extinction Recall	CS: Three lamps US: Electric shock	SCR-fMRI	Cortisol group showed reduced activations in the bilateral amygdala, right anterior parahippocampal gyrus, and right hippocampus during extinction training
Hagedorn et al. [117]	Cortisol group (30) Placebo group (30)	10 mg of cortisol Placebo	Cortisol is an agonist of glucocorticoid receptor and Annexin A1	Acquisition Extinction Recall Reinstatement	CS: Geometric figures US: Electric shock	SCR-fMRI	Stimulus-based extinction generalization increased fear-related brain activation and altered functional connectivity during both extinction learning and recall, but these effects were reversed by cortisol administration
Hagedorn et al. [119]	Cortisol group (30) Placebo group (30)	20 mg of cortisol Placebo	Cortisol is an agonist of glucocorticoid receptor and Annexin A1	Acquisition Extinction Recall Reinstatement	CS: Geometric figures US: Electric shock	SCR-fMRI	Cortisol prior to extinction generalization improved the extinction memory during the reinstatement test
Brueckner et al. [118]	Cortisol group (25) Placebo group (25)	30 mg of cortisol Placebo	Cortisol is an agonist of glucocorticoid receptor and Annexin A1	Acquisition Extinction Reinstatement	CS: Images of neutral male/female faces US: Traumatic clips	SCR-FPS	Cortisol group showed less reinstatement, lower US expectancy for the CS+, and attenuated FPS for the CS+, as compared with the placebo group

**Table 2:** Summary of findings in studies that explore the effects of cortisol and sex on fear extinction.

Study	Group (N)	Pharmacological Treatment	Mechanism of Action	Phase of Fear Learning	CSs	Psychophysiological Measure	Main Findings
Stark et al. [66]	Female cortisol (8) Male cortisol (9) Female placebo (9) Male placebo (8)	30 mg of cortisol Placebo	Cortisol is an agonist of glucocorticoid receptor and Annexin A1	Acquisition	CS: Geometric figures US: Electric shock	SCR-fMRI	Cortisol abolished the enhanced first interval responses for CS+ in men but increased it in women Men, but not women, in the placebo group showed stronger response to the CS+ than to the CS−
Merz et al. [134]	Female cortisol (10) Male cortisol (10) Female placebo (9) Male placebo (10)	30 mg of cortisol Placebo	Cortisol is an agonist of glucocorticoid receptor and Annexin A1	Acquisition Extinction	CS: Geometric figures US: Electric shock	SCR-fMRI	Cortisol reduced SCRs to CS in women and enhanced SCRs to both CS in men, compared to the placebo group Cortisol reduced amygdala reactivity to the CS+ both in men and women, but enhanced activity in the right insula only in women
Merz et al. [135]	LU women (15) OC women (15) Men group (20)	30 mg of cortisol Placebo	Cortisol is an agonist of glucocorticoid receptor and Annexin A1	Acquisition Extinction	CS: Geometric figures US: Electric shock	SCR-fMRI	Men showed higher SCRs to CS+ compared to OC women, whereas LU women did not significantly differ from men LU women showed higher CS+/CS− differentiation in the right amygdala compared to OC women and men
Meir Drexler et al. [136]	Reactivation + cortisol (14) Reactivation + placebo (14) No reactivation + cortisol (14)	30 mg of cortisol Placebo	Cortisol is an agonist of glucocorticoid receptor and Annexin A1	Acquisition Memory reactivation Extinction Reinstatement	CS: Geometric figures US: Electric shock	SCR	RE + CORT group only had higher SCRs for the reactivated CS1+ in the reinstatement
Meir Drexler et al. [137]	Reactivation + cortisol (24) Reactivation + placebo (24) No reactivation + cortisol (24)	30 mg of cortisol Placebo	Cortisol is an agonist of glucocorticoid receptor and Annexin A1	Acquisition Memory reactivation Extinction Reinstatement	CS: Geometric figures US: Electric shock	SCR	No differences in the reinstatement of the three CSs in any of the groups
Meir Drexler et al. [138]	Reactivation + cortisol (25) Reactivation + placebo (25) No reactivation + cortisol (25)	20 mg of cortisol Placebo	Cortisol is an agonist of glucocorticoid receptor and Annexin A1	Acquisition Memory reactivation Extinction Reinstatement	CS: Geometric figures US: Electric shock	SCR	No reactivation group had higher SCRs for the reactivated CS1+ in the reinstatement No reinstatement effect was found in the two reactivation groups, regardless of the pharmacological treatment

## Data Availability

Not applicable.

## Abbreviations

NS	neutral stimulus
US	unconditioned stimulus
CS+	conditioned stimulus
CS−	conditioned stimulus never paired with the US
ROF	return of fear
SCR	skin conductance response
EMG	electromyography
fMRI	functional magnetic resonance imaging
ROIs	regions of interest
BOLD	blood-oxygen-level-dependent
PTSD	post-traumatic stress disorder
HPA	hypothalamic–pituitary–adrenal axis
GRs	glucocorticoid receptors
DEX	dexamethasone
HC	hydrocortisone
FPS	fear-potentiated startle
BMI	body mass index
vmPFC	ventromedial prefrontal cortex
dACC	dorsal anterior cingulate cortex
RE + CORT	reactivation + cortisol
RE	reactivation
CORT	cortisol
SPM	statistical parametric mapping
